# Rehabilitation progress following reverse total shoulder replacement and internal fixation for geriatric three and four-part proximal humerus fractures – a propensity score matched comparison

**DOI:** 10.1186/s12891-023-06669-3

**Published:** 2023-07-11

**Authors:** Chi Him Tong, Christian Xinshuo Fang

**Affiliations:** 1 Department of Orthopaedics & Traumatology, Queen Mary Hospital, Hong Kong, Hong Kong; 2 Department of Orthopaedics & Traumatology, The University of Hong Kong, Hong Kong, Hong Kong

**Keywords:** Geriatric fracture, Proximal humerus fracture, Reverse shoulder arthroplasty, Open reduction internal fixation, Rehabilitation progress

## Abstract

**Background:**

Proximal humerus fracture is one of the most common fractures in the elderly population. However, in patients with complex fracture patterns, there is still no general consensus in the best treatment method. This study aims to evaluate the outcomes between those treated with reverse total shoulder arthroplasty (rTSA) and open reduction internal fixation (ORIF).

**Methods:**

All geriatric patients (> 60 years of age) with proximal humerus fractures undergoing surgical treatment were analysed. There were 25 patients treated with rTSA and 75 with ORIF. Propensity score matching was used to select 25 matching patients from the ORIF group according to age and gender. All patients underwent surgical intervention within 7 days (mean 3.8 days). All patients followed a protocol-driven rehabilitation programme with outcome assessment at 3, 6, 12 and 24 months. Constant score, qDASH, range of motion, rate of complications and revision surgery were recorded and compared.

**Results:**

Twenty-five rTSA were age and gender matched with 25 ORIF patients. The average age of patients in rTSA and ORIF groups were 77.0 years and 75.2 years respectively. At 3 months, mean Constant score was 37.7 (rTSA) vs 45.5 (ORIF) (*p* = 0.099). Mean qDASH score was 50.6 (rTSA) vs 29.4 (ORIF) (*p* = 0.003). Mean forward flexion range was 72.9° (rTSA) vs 94.4° (ORIF) (*p* = 0.007). Mean abduction range was 64.0° (rTSA) vs 88.6° (ORIF) (*p* = 0.001). At 2 years, mean Constant score was 72.8 (rTSA) vs 70.8 (ORIF) (*p* = 0.472). Mean qDASH score was 4.50 (rTSA) vs 11.0 (ORIF) (*p* = 0.025). Mean forward flexion range was 143° (rTSA) vs 109° (ORIF) (*p* < 0.001). Mean abduction range was 135° (rTSA) vs 110° (ORIF) (*p* = 0.025). There was a higher number of complications observed for ORIF (3) than rTSA (1) (*p* = 0.297) and a higher number of re-operations for ORIF (3) than rTSA (1) (*p* = 0.297), which was not statistically significant.

**Conclusion:**

rTSA appears to yield a slower recovery at 3 months but a better outcome at 2 years. It is a promising treatment for geriatrics with three- and four-part proximal humerus fractures aiming for a better long-term functional outcome.

## Introduction

In the modern aging society, the incidence of osteoporosis and fragility fractures continues to increase [1, 2]. Proximal humerus fracture is the third most common non-vertebral fracture pattern seen in the geriatric population [3], which causes major morbidity to the elderly [4] in terms of pain and function, as well as a significant burden to the health care system [5]. The incidence is estimated to be tripled after 30 years [6].

Although the operative indications of these fractures are still poorly defined [7], surgeons have been treating them increasingly by operative means in recent years [8], as influenced by a number of factors, such as patient’s age, severity of the fracture and presence of glenohumeral dislocation [9].

High-grade fractures, such as 3-part and 4-part fractures in the Neer classification system [10], have been traditionally associated with worse outcomes from factors such humeral head ischemia and tuberosity failures [11], which has led to the debate of treating these patterns.

The treatment of geriatric proximal humerus fracture has evolved a lot in the modern era. Factors such as patient’s age, severity of the fracture and presence of glenohumeral dislocation [9], all play a role in surgeon’s decision. Geriatric fractures are considerably more challenging to treat surgically given the high prevalence of osteoporosis and poor rotator cuff status. Currently, the most common surgical modalities include open reduction internal fixation (ORIF) with locking plates [12, 13], intramedullary nailing, hemiarthroplasty [14], and reverse total shoulder arthroplasty [15, 16]. However, there is still insufficient evidence and no consensus regarding which is the best surgical option [17] as each has its own drawback. The functional outcome is influenced by the healing status of the tuberosity [18], the conditions of the rotator cuff [19], and the need for prolonged immobilization after surgery [20], which is not desirable in the elderly.

Reverse total shoulder arthroplasty (rTSA) is becoming the treatment of choice among surgeons for 4-part fractures [21], and the number of operations performed has increased over the past decade [22] and providing favourable mid to long term outcomes compared to hemiarthroplasty [23–27] and ORIF [28, 29]. On the other hand, there are also suggestions that reverse total shoulder arthroplasty may cause more complications than traditional ORIF [30].

Most of the above studies focus on the long-term outcome but research on enhancing early functional recovery is lacking. In all types of surgery, immediate mechanical stability without need protect repaired structures allows early mobilization and enhances recovery. However, since replacement is seen as a more major undertaking with more soft tissue dissection compared to fixation, it is uncertain which option provides a speedier recovery. We hypothesize a difference in early and late recovery, since fixation is more anatomical, provides immediate stability to allow quicker short-term recovery, while replacement requires retraining the deltoid to substitute cuff function, which may improve function better in the long run.

The purpose of this study is to evaluate and compare the early and mid-term rehabilitation progress in geriatric patients with 3-part and 4-part proximal humerus fractures after rTSA versus ORIF.

## Materials and methods

### Study design

Consecutive patients presenting with proximal humerus fracture from 2015–2020 were identified from a local registry admitted to a single university-affiliated centre. During this period, patients aged > 60 with proximal humerus fractures receiving surgical treatment either by ORIF with Locking Compression Plate (LCP) or rTSA were included. Patients not having a three- or four-part proximal humerus treated surgically, not following a standardized rehabilitation protocol, those with pathological fractures, and those having less than 2 years of follow-up were excluded. Patients were divided according to their surgical treatment into two groups, namely rTSA and ORIF, respectively.

To match the patients between the two groups and control for confounding factors, we used the propensity score matching method with the nearest neighbour technique with a caliper of 0.2. Patients were matched with baseline factors – age and gender. There were 25 patients who have undergone rTSA, and 25 patients with ORIF were selected from a total of 75 using this method. The two groups were compared in terms of functional outcomes, surgical complications and revision surgery.

Every patient received standard shoulder radiographs (AP and Scapular Y views) and a computer tomography (CT) scan before the operation. Using the Neer’s classification [10] based on the pre-operative CT scan, we define one part as a fracture fragment with either displacement more than 1 mm or angulation more than 45 degrees.

In our centre, patients with non-reconstructible fractures were treated with rTSA, including head-split fractures, anatomical neck fractures, highly comminuted 4-part fractures, and associated glenohumeral dislocation. Indications for ORIF include young age, reparable tuberosity and reconstructible fracture patterns. Indications for non-operative treatment include minimally displaced fractures, valgus impacted fractures, tuberosity displacement less than 5 mm, and neck-shaft angle within 10 degrees of normal [9].

### Surgical technique – rTSA group

In the rTSA group, 21 patients received Delta Xtend prosthesis (DePuy Synthes, Indiana USA) and 4 patients received Aequalis II prosthesis (Tournier, Grenoble France). In all patients, a 155-degree cemented stem and a glenosphere with diameter of 36–38 mm was used.

Standard anterior deltopectoral approach was adopted in all patients. Implants include a cementless glenoid baseplate of 25–27 mm fixed with 2–4 glenoid screws of variable length, a 155-degree cemented humeral stem, and a polyethylene insert. All patients had greater tuberosity repaired to the humeral stem with 4-strand braided non-absorbable Orthocord suture (DePuy Synthes).

### Surgical technique – ORIF group

The PHILOS System (DePuy Synthes, Indiana USA) is an anatomical locking compression plate, designed for proximal humerus fixation. It allows minimally invasive plate osteosynthesis (MIPO) with locking screw fixation, which is preferable for geriatric osteoporotic bone. Surgeons have a number of options for proximal and distal fixation, increasing the overall stability of the construct.

Split-deltoid approach was used in all patients. Fracture reduction was confirmed with fluoroscopic guidance. The PHILOS plate was inserted with MIPO approach [31] proximal to the level of the axillary nerve, with at least 5 proximal screws and at least 3 distal screws inserted via a separate incision distally. Non-absorbable sutures were used to augment the supraspinatus and infraspinatus to the PHILOS plate.

### Outcomes

All patients (rTSA and ORIF) followed a protocol-driven rehabilitation program jointly by surgeons, physiotherapists and occupational therapists in a designated rehabilitation unit. At week 0–4, all patients started on gentle passive mobilization to facilitate fracture & tuberosity healing. At week 4 onwards, patients were allowed free active and passive mobilization, and progressive strengthening as tolerated if there was radiological evidence of healing. Clinical follow-ups and outcome assessment performed at post-operative 3, 6, 12, 24 months. Outcomes including Constant score, qDASH score, range of movement, surgical complications and any need for revision surgery, were recorded and compared between the two groups. Complications in rTSA were defined as acromion fractures, dislocation, neurovascular injuries, periprosthetic joint infection and component loosening. Complications in ORIF group were defined as screw penetration into the joint, loss of reduction or fixation, tuberosity displacement and avascular necrosis.

### Statistical analysis

Statistical analysis was made with SPSS software (version 27, IBM, Armonk, USA). Propensity score matching was carried out with the Thoemmes plugin [32] using nearest neighbour calliper of 0.2 with age and gender as the confounding factors. Categorical variables were compared with chi-squared test, while continuous variables were presented in the form of mean ± standard deviation, and compared with independent t test, and *p*-value of < 0.05 was considered statistically significant.

## Results

### Patient groups

During the study period, we have treated 330 consecutive proximal humerus fractures in patients aged > 60, 25 patients underwent rTSA, and 116 had ORIF with Locking Compression Plate (LCP). The 25 patients who underwent rTSA were matched using propensity score against 25 patients in the ORIF which 75 with ORIF had 2-years follow-up. Patients in rTSA group were operated by a single surgeon, while those who underwent ORIF were operated amongst 3 experienced surgeons, all specialized in orthopaedic trauma. In the rTSA group, 1 patient died between 1-year and 2-year, and 2 patients were lost to follow-up between 1-year and 2-year. In the ORIF group all patients reached their 2-year follow-up.

The patient’s baseline demographics are listed in Table 1 below:

**Table 1:** Baseline demographics

	rTSA (*n* = 25)	ORIF (*n* = 25)	*p*-value
Gender	M = 3	M = 3	
	F = 22	F = 22	
Mean age at operation	77.0 ± 7.14	75.2 ± 6.79	*p* = 0.361^
Fracture pattern	3-part = 6^a^	3-part = 22	*p* < 0.001*
	4-part = 19	4-part = 3	

^a^Among the six 3-part fractures in rTSA group, 5 involve anatomical neck and 1 is head-split fracture

^*^Chi-squared test

^Independent t-test

The mean surgical duration and hospital stay of the 2 groups are compared and summarized in Table 2.

**Table 2:** Surgical duration and hospital stay

	rTSA (*n* = 25)	ORIF (*n* = 25)	*p*-value
Mean surgical duration (mins)	133 ± 52.2	89.7 ± 25.0	*p* < 0.001^
Mean length of hospital stay (days)	20.1 ± 17.0	9.92 ± 6.49	*p* = 0.009^

^Independent t-test

### Functional outcomes

Overall, rTSA appears to yield worse functional outcomes before 6 months but better functional outcomes at 2 years. Consistently, the rehabilitation of rTSA underperforms ORIF before 6 months but this pattern is reversed at 12 and 24 months except for external rotation and internal rotation range. The average Constant shoulder score, qDASH score, forward flexion range, abduction range, external rotation range and internal rotation range are compared and summarized in Table 3.

**Table 3:** Functional outcomes

	rTSA (*n* = 25)	ORIF (*n* = 25)	*p*-value^
Constant score			
3 month	37.7 ± 15.6	45.5 ± 11.5	*p* = 0.099
6 month	55.2 ± 13.7	60.0 ± 11.1	*p* = 0.290
12 month	68.1 ± 10.8	64.6 ± 11.7	*p* = 0.403
24 month	72.8 ± 5.56	70.8 ± 10.1	*p* = 0.472
qDASH score			
3 month	50.6 ± 12.8	29.4 ± 18.2	*p* = 0.003
6 month	18.2 ± 15.8	18.5 ± 14.1	*p* = 0.957
12 month	11.0 ± 7.98	18.4 ± 16.6	*p* = 0.242
24 month	4.50 ± 3.63	11.0 ± 7.63	*p* = 0.025
Forward flexion range (degrees)			
3 month	72.9 ± 25.7	94.4 ± 16.7	*p* = 0.007
6 month	104 ± 31.9	108 ± 16.7	*p* = 0.591
12 month	136 ± 27.8	109 ± 22.6	*p* = 0.006
24 month	143 ± 21.5	109 ± 23.8	*p* < 0.001
Abduction range (degrees)			
3 month	64.0 ± 26.4	88.6 ± 23.2	*p* = 0.001
6 month	105 ± 35.4	106 ± 22.7	*p* = 0.890
12 month	125 ± 33.4	108 ± 27.0	*p* = 0.095
24 month	135 ± 28.1	110 ± 29.6	*p* = 0.025
External rotation range score ^a^			
3 month	2.29 ± 3.67	3.63 ± 2.93	*p* = 0.224
6 month	3.62 ± 2.93	5.80 ± 2.70	*p* = 0.034
12 month	5.33 ± 3.61	6.14 ± 3.09	*p* = 0.536
24 month	6.00 ± 3.06	6.20 ± 3.49	*p* = 0.894
Internal rotation range score ^a^			
3 month	2.86 ± 3.02	3.92 ± 2.32	*p* = 0.329
6 month	4.14 ± 3.08	6.16 ± 1.99	*p* = 0.045
12 month	6.00 ± 1.41	6.91 ± 1.82	*p* = 0.308
24 month	8.00 ± 0.00	7.50 ± 1.82	*p* = 0.234

^a^External rotation and internal rotation range is scored on scale of 10 based on the objective assessment component in the Constant score

^Independent t-test

**Fig. 1:**
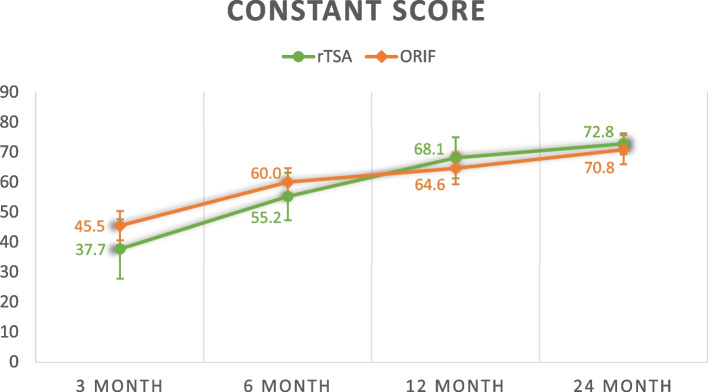
Constant score

**Fig. 2:**
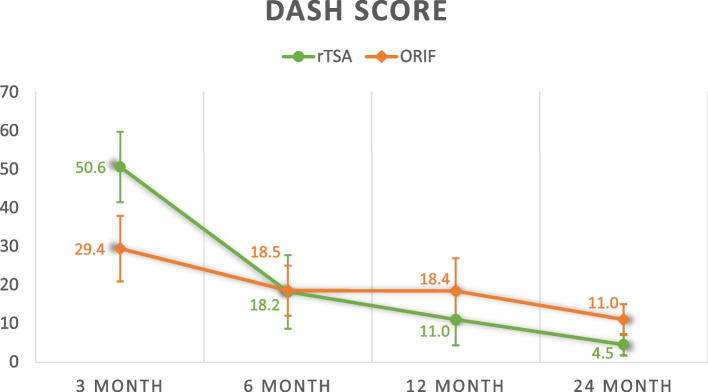
Dash score

**Fig. 3:**
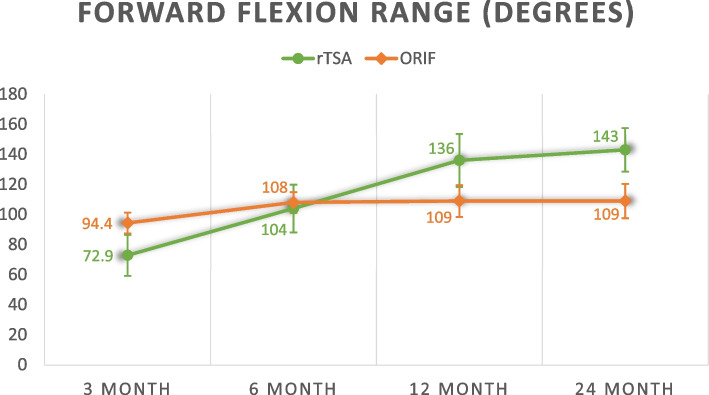
Forward flexion range

**Fig. 4:**
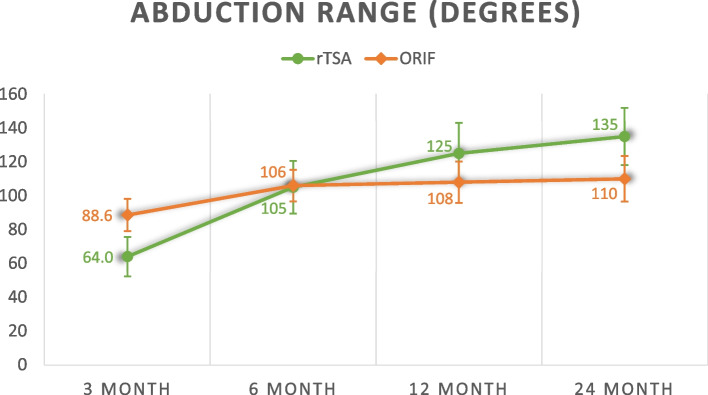
Abduction range

**Fig. 5:**
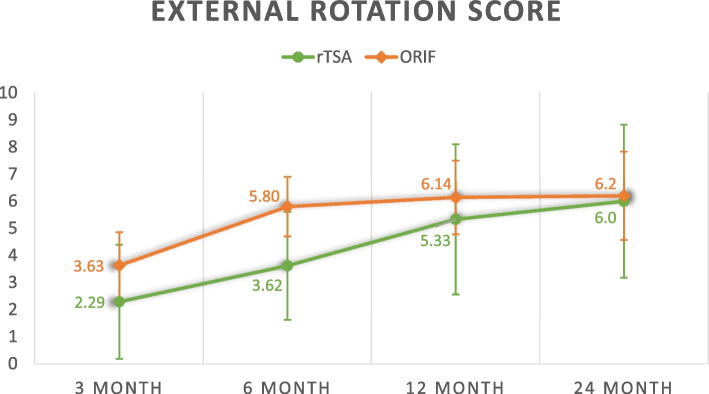
External rotation score

**Fig. 6:**
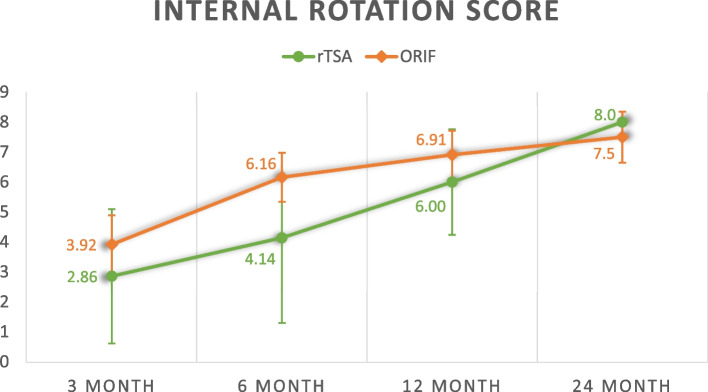
Internal rotation score

The following figures (Figs. 1, 2, 3, 4, 5 and 6) compare the functional score and range of motion between the rTSA group and the ORIF group. The center dots represent the mean, while the vertical bars represent the 95% confidence interval.

### Radiological outcomes

In the rTSA group, there were 3 patients observed to have scapular notching (all Grade 1 according to the Nerot-Sirveaux classification), 5 patients with tuberosity non-union, but none had signs of mechanical loosening or dislocation. In the ORIF group, immediate post-operative radiograph showed all fractures were well reduced with neck-shaft angle less than 10 degrees deviation from normal. All patients achieved radiographic union.

### Complications

There were more complications observed for ORIF (3) than rTSA (1), *p* = 0.297. In the rTSA group, 1 patient developed post-operative wound infection, which settled after debridement, antibiotics and retention of implants. Grade 1 scapular notching was not regarded as a complication as it was not associated with functional impairment or loosening. None of them had radiological evidence of mechanical component loosening or dislocation. In the ORIF group, 3 patients had screw penetration into the shoulder, in which 2 of them developed osteonecrosis of the humeral head. No patient had loss of fixation. No axillary nerve injury was reported.

### Reoperations

There were more reoperations after ORIF (3) than rTSA (1), *p* = 0.297. 1 patient who developed post-operative wound infection in rTSA group was reoperated for debridement; the prosthesis was not loosened and was thus retained. In the ORIF group, 3 patients who screw protrusion into the joint subsequently underwent removal of implants. The mean duration from index operation to removal of implants is 6 months. All 3 patients already had fracture union at the time of removal.

## Discussion

We observed from our study that the improvement rate of shoulder function and range of motion is slower in rTSA before 6 months versus ORIF. However, patients with rTSA had sustained improvement and attain better function and range than ORIF at 2 years. The “flip effect” at 6 months is an interesting phenomenon, at which the rTSA group started to surpass the ORIF group in terms of functional score and range. We also observed a higher rate of complications and reoperations in the ORIF group, although it was not statistically significant.

The goals of treating geriatric proximal humerus fractures include optimizing pain relief, providing a stable construct for early rehabilitation, as well as minimizing complications and need for secondary surgical intervention. Current literature shows the most common surgical options include ORIF with locking plate, hemiarthroplasty and rTSA all being reasonable options, albeit each has its own benefits and specific risks.

The Locking Compression Plate (LCP) fixation of osteoporotic bone with Minimally Invasive Plate Osteosynthesis (MIPO) has shown promising clinical results [33, 34] in most fractures. However, LCP fixation is not without risks. Hardeman [35] et al. evaluated 307 shoulders and reported an overall 15.3% failure rate and 23.8% re-operation rate at 4.3 years. The most common complications were screw penetration, loss of reduction, and avascular necrosis [36, 37]. Screw penetration often requires a revision procedure, while loss of reduction primarily occurs in the presence of varus malreduction [12, 37]. Anatomical reduction and restoration of medial cortical support [38] are essential for successful surgical fixation [39], which can be difficult in comminuted fractures. Humeral head osteonecrosis is also a risk. As a result, some surgeons suggest primary shoulder arthroplasty as an option for complex proximal humerus fractures.

Primary shoulder hemiarthroplasty is an accepted option for complex proximal humerus fractures [14], but its success depends on several factors. While it can provide satisfactory pain relief, restoration of shoulder range of motion and function is less predictable [14, 40, 41]. The healing of tuberosities at the anatomical location is critical to post-operative function and range of motion [42, 43]. Problem with tuberosity healing is observed radiologically in 11% of patients [41] and only half of patient may attain shoulder abduction of above 90 degrees [44]. Patients' age and type of prosthesis used are also influential factors in the success of the procedure. Moreover, even in pre-injured and asymptomatic shoulders, the prevalence of rotator cuff tears is significant and correlates positively with age [19, 45, 46]. Surgeons should be prepared to convert to other forms of shoulder arthroplasty if rotator cuff tears are found during the surgery.rTSA is a preferred surgical treatment of late stage cuff tear arthropathy, which medializes center of rotation, lengthens the deltoid muscle and increases the deltoid lever arm [47]. It’s advantage of being independent to rotator cuff status [48] is applicable to the treatment of proximal humerus fractures [21, 22, 49]. Risk of dislocation, glenoid component loosening and scapular notching [50, 51] is less common with improved prosthesis design and technique avoiding superior placement of glenospheres [52], and careful reattachment of the tuberosities [53]. In all, functional outcome of rTSA is more reliable than hemiarthroplasty and the rate of complications appears to be low.

The 2-year rTSA outcome of this study is similar to several others in the literature. Bufquin et al. [54] evaluated 43 shoulders with mean follow-up of 22 months after rTSA. The mean Constant and the mean modified Constant scores were 44 and 66% respectively. The mean active anterior elevation was 97 degrees and the mean active external rotation in abduction was 30 degrees. Longo et al. [55] reviewed 256 patients with mean follow-up of 27.8 months after rTSA. Overall, the mean Constant score was 56.7 ± 7.6 points, the mean DASH score was 39.9 ± 6 points. More recently, Fitschen-Oestern et al. [56] evaluated 23 shoulders with mean follow-up of 28.4 months after rTSA. The mean Constant score was 55 ± 13, while the mean shoulder abduction range was 111 degrees, while the external rotation in abduction was 25.87 degrees. However, none of the above papers showed comparison between rTSA and ORIF in term of early rehabilitation progress, which is important in managing patient’s expectations in the early post-operative period.

Simovitch et al. [57] described the post-operative rate of improvement of rTSA – full improvement was achieved by 24 months, although the majority of improvement was achieved in the first 6 months. However, ORIF has even quicker recovery in very early post-operative phase, given less surgical trauma, surgical duration and length of hospital stay. Merschin et al. [58] showed significant benefit of rTSA at improving health-related quality of life. The DelPhi trial [59] showed better 2-year Constant score in rTSA compared with ORIF for patients with AO Type C2 fractures. The strength of the study lies in its randomized design, however its limitations include uncertainty regarding radiological reduction and implant placement quality. Our results showed comparable Constant score in the rTSA group at all time intervals consistently, though our results in the ORIF group performed considerably better than those in the DelPhi trial. A recent study by Lanzetti et al. [60] also showed comparable long-term results to our study, with rTSA performing better in terms of Constant score, DASH score, elevation and abduction range. However, it did not evaluate the short-term outcomes, like our study did.

We showed than complex fractures treated with ORIF have worse functional outcome than rTSA from 6 months onwards. Possible ways to improve include maintenance of training until 2 years post-operatively, as well as to employ individualized training – identify the patient’s difficulties in handling specific tasks in daily living or occupation, and train accordingly. Complications are also not uncommon following ORIF, namely screw penetration, loss of reduction and avascular necrosis. Frequent clinical and radiological assessment and early recognition of these adverse events are essential to optimize patient’s long term function.

This study has its own limitations. Firstly, a retrospective study is prone to selection bias. The ORIF group predominantly consists of 3-part fractures while the rTSA group predominantly consists of 4-part fractures. Theoretically, rTSA following a 3-part or 4-part fractures should not make much of a difference. Secondly, no randomization was performed. However, propensity score was matched between the two groups to limit confounding factors. Thirdly, a small sample size (25 rTSA vs 25 ORIF) limits the statistical power of our study.

This is the first study that provides an in-depth analysis on the early rehabilitation progress between the two different treatment groups. The strength of this study includes a direct comparison between the two groups, with propensity scored matching to control for confounders. All patients were follow-up for a minimum of 2 years with documentation of clinical scores. A single-centred design may minimize variability in inclusion, surgical technique, rehabilitation and outcome measurement.

## Conclusion

Compared with internal fixation, rTSA appears to yield a slower recovery before 6 months but a better functional outcome at 2 years, and also has an overall lower complication rate. rTSA is a promising treatment for three- and four-part proximal humerus fractures in geriatric patients aiming for a better long-term functional outcome.

## Data Availability

The data that support this study are available from the Hong Kong Hospital Authority Clinical Data and Reporting System (CDARS), but restrictions apply to these data, which were used under license for the current study, and so are not publicly available. Data however are available from corresponding author (Dr. CH Tong) upon reasonable request.
